# Design, validation and implementation of the post-acute (long) COVID-19 quality of life (PAC-19QoL) instrument

**DOI:** 10.1186/s12955-021-01862-1

**Published:** 2021-09-28

**Authors:** Ravi Jandhyala

**Affiliations:** 1 Medialis Ltd, 13 Horse Fair, Banbury, OX16 0AH UK; 2 Faculty of Life Science and Medicine, Centre for Pharmaceutical Medicine Research, Institute of Pharmaceutical Science, King’s College London, Franklin Wilkins Building, 150 Stamford Street, London, SE1 9NH UK

**Keywords:** COVID-19, Public health, Quality of life, Statistics, Research methods

## Abstract

**Background:**

The novel coronavirus (SARS-CoV-2) has led to a global pandemic, resulting in a disease termed COVID-19, which commonly presents in adults as a typical infection of the upper respiratory tract. Although the disease is often acute, one in ten patients can continue to be affected for weeks or months, resulting in a state called long COVID. Existing evidence suggests there are no patient-centred instruments for capturing the impact of long COVID on the quality of life of people affected.

**Methods:**

The Jandhyala Method was used to identify indicators of long COVID quality of life. The resulting post-acute (long) COVID-19 Quality of Life (PAC-19QoL) instrument was validated with a control group of unaffected participants and finally implemented in the dedicated patient registry, PAC-19QoLReg.

**Participants:**

15 participants suffering from long COVID, who have been positively diagnosed with COVID-19, either via diagnostic or antibody tests and a validation control group of 16 healthy participants who have not suffered from COVID-19.

**Main outcome measures:**

Indicators submitted by participants with long COVID that address the specific impact of the illness on their quality of life.

**Results:**

Forty-four Quality of Life Indicators (QoLI) across four domains, namely, psychological, physical, social, and work, were agreed by the participants with long COVID to be relevant for the assessment of their quality of life (CI > 0.5). The validation stage identified 35/44 QoLIs that differentiated between the two groups, with a statistically significant difference between the mean QoLI Likert Scores (*p* < 0.05).

**Conclusions:**

The PAC-19QoL instrument and PAC-19QoLReg prospective observational cohort clinical study will enable an understanding of disease progression, on and off treatment, on the quality of life of patients with long COVID beyond simple symptomatology.

*Trial registration*: ClinicalTrials.gov Identifier NCT04586413; 14th October 2020.

## Background

From December 2019 onwards, a novel coronavirus (SARS-CoV-2) spread worldwide. In March 2020, the World Health Organization classified it as a pandemic [1, 2]. The resulting disease from infection with SARS-CoV-2, termed COVID-19, commonly presents in adults as a typical infection of the upper respiratory tract. It ranges from mild to moderate fever, cough and fatigue [3, 4]. In severe cases, pneumonia may develop (15% of cases), whilst an estimated 5% of COVID-19 patients suffer from acute respiratory distress syndrome (ARDS), septic shock and/or multiple organ failure [5, 6]. Furthermore, it is anticipated that COVID-19 may have a major impact on physical, cognitive, mental and social health status, even in patients with mild disease presentation [7].

Although the disease is often acute, one in ten patients can continue to be affected for weeks or months [6]. This so-called "long COVID" can result in extreme fatigue, muscle and joint pain, breathlessness, heart palpitations, loss or alteration of the sense of taste and smell, gastrointestinal distress and problems with attention, memory and cognition [6]. In a continued pandemic state, this is likely to contribute significantly to global morbidity and mortality. However, the majority of COVID-19 research has been focused on the pathogenesis of SARS-CoV-2 and therapeutic strategies, such as vaccines or antiviral drugs [8]. Consequently, there is a lack of formal evidence of any long COVID impact on both the health and quality of life of affected individuals. This represents an important evidence gap [8].

Research into COVID-19 has preferentially focussed on symptomology, the acute nature of this illness and involved interventions such as therapeutics and vaccines. However, many patients display persistent symptoms with a continued impact on their quality of life weeks and months after the initial disease. Research on the impact of long COVID on quality of life is scarce and, until now, without a validated disease-specific instrument.

Current tools being developed, such as a clinician facing prognostic communication tool with COVID-19 and critical illnesses [9], appear to be clinician-focused. As such, they fail to address the potential impact of COVID-19 on patients' lives beyond its symptoms. Furthermore, these tools are not sufficient to capture QoL, as they lack the sensitivity to capture the often complex and multi-dimensional nature of QoL and its changes [10]. The same is true of non-disease-specific QoL instruments being used in preference to disease-specific ones due to the lack of neutrality of the indicators used [11–13].

Hence, this necessitates the development of a patient-centred long COVID specific quality of life instrument. As with other diseases, for one reason or another, the majority of this disease population lie beyond the reach of a clinical trial. Thus, researching this real-world population continues to be of critical importance to a range of interested parties. This includes regulators, payors and prescribers. Their vehicle of choice is the patient registry or observational cohort clinical study of a prospective and/or retrospective type [14].

### Study aim and objectives

The overarching aim of this project is to create a disease-specific (long COVID) quality of life instrument to complement existing tools and ongoing initiatives geared towards improving the quality of life of people affected by COVID-19.

#### Objectives

Hence, the objectives of this research are to:

1. design a post-acute COVID-19 Quality of Life (PAC-19QoL) instrument for the assessment of individuals with long COVID.
2. Validate the developed PAC-19QoL instrument using healthy volunteers.
3. Implement the developed PAC-19QoL instrument as the core dataset in the associated PAC-19QoLReg patient registry (ClinicalTrials.gov Identifier: NCT04586413). The patient registry is currently recruiting participants with a clinical diagnosis of long COVID irrespective of test confirmation for a 12-month follow-up period.

## Methods

### Study participants

Recruitment took place in December 2020 from adverts placed on social media sites, informal online support groups and snowballing via recruited subjects promoting the study within their own networks of long COVID sufferers. A total of 15 participants, who had either a diagnostic or antibody test confirmation for SARS-COV-2 and were still suffering from post-acute symptoms of COVID-19, were recruited to the study group. Sixteen healthy volunteers participated in the study as the control group to validate the developed PAC-19QoL instrument.

### Sample size

The sample size calculation was informed by the need to both achieve saturation of PAC-19QoL indicators and assess the degree to which each indicator differentiated between the disease and non-disease state. Previous experience with the Jandhyala Method, predicted saturation of unique indicators at a minimum target sample size of 10 with an upper limit of 20. For validation, using a univariate approach, an indicator was deemed to differentiate between the two groups if the prevalence of the indicator was > 50% in one group than the other. A minimum sample size of 15 participants per group was calculated to provide sufficient power (at least 80%) to detect this difference of 50% between the two groups at a 5% level of significance.

### Identification of PAC-19QoL quality of life indicators (variables) and summary of the Jandhyala method

Using the Jandhyala Method, the PAC-19QoL indicators (QoLIs), also referred to as variables, were identified [15]. The Jandhyala Method is a novel method for observing proportional group awareness and consensus on responses arising from a list-generating questionnaire. The method has been differentiated from competing consensus generating methodologies [16]. The Jandhyala Method has been validated against Delphi, non-Rand modified Delphi and Rand Appropriateness Method in a systematic literature review and was found to be unique in observing consensus and measuring awareness of subject matter across experts. The Jandhyala Method also improves upon the traditional Delphi-style methodologies, through the introduction of new insights into awareness of subject matter in the expert group. It employs a highly efficient two-round anonymised survey approach without any face-to-face interactions between experts.

The participant consensus is achieved by observing levels of awareness and consensus relating to a list of recommended QoLIs for PAC-19QoL. These are solicited via two anonymised online surveys and calculating an awareness index (AI) and consensus index (CI) for each item, respectively. The responses to the awareness round questionnaire were used to assess knowledge awareness by calculating the frequency of each coded item in relation to the overall most frequently occurring coded item (the Awareness Index). Consequently, the consensus index is measured as the percentage of participants supporting the included item, indicating agree or strongly agree to the included item.

The AI and CI, both continuous variables, were further categorised into 4 Awareness and Consensus scores: Complete Awareness or Consensus (AI or CI = 1.00) – A1 or C1; Awareness/ Consensus + (0.50 < AI or CI) – A2 or C2; Consensus – (0 < AI or CI ≤ 0.50) – A3 or C3; and No consensus (AI or CI = 0) – A4 or C4 [15].

### Operationalisation of the Jandhyala method in this study

During the first Awareness Round (1) survey, participants were asked to respond to a series of demographic questions, and two main list-generating questions. Participants were asked to provide a minimum of three and a maximum of ten free-text answers. Please refer to Appendix 1 for the instructions given to the Awareness Round (1) survey participants.

The participants' responses from this Awareness Round (1) were analysed per group. They were then refined into a mutually exclusive list of quality of life indicators by three researchers using a process of content analysis and open coding [17, 18]. The codes were then attributed to the relevant participants' answers by one researcher and were confirmed by a second researcher.

The participants who completed the first round were asked to participate in the second Consensus Round (2) survey. They were asked to rate their level of agreement with the inclusion of the QoLIs arising from the Awareness Round (1) survey, using a five-point Likert scale (Strongly agree, Agree, Neither agree nor disagree, Disagree and Strongly disagree). Quality of Life indicators reaching a consensus level of > 50% (CI > 0.5) were retained in the final list and used to populate the PAC-19QoL.

### Variables: operational definition and scale

Following the identification of the quality of life indicators, these were included in the PAC-QoL survey instrument as the variables. Study participants were required to rate the variables (QoLIs) using the Likert Scale. The variables included in the PAC-19QoL survey instrument, and their operational definitions are shown in Table 2.

### Statistical analyses

#### Demographics

Using a Chi-square test, the binary presence or absence of the following discrete characteristics were compared between the control and study groups: sleep apnoea, sleeping difficulty, staying asleep difficulty, allergies, assistance with self-care needs, cancer diagnosis, current smoker, doing own shopping, difficulty falling asleep, former smoker, gender, immunosuppressant drugs, long COVID in the past, long COVID symptoms, major surgeries, mobility issues, non-prescribed or homoeopathic medications, organ transplant, other family history condition, physical activity, pregnancy weeks, prescribed medications**.**

The following categorical increasing grades of pre-existing conditions were compared between both groups using a Chi-square test: "no versus mild/moderate/severity" (asthma, diabetes, high blood pressure, cholesterol, chronic obstructive pulmonary disease (COPD), cystic fibrosis, stroke). Due to the type of statistical analysis conducted, weight categories were compared "underweight/average" versus "obese", ethnicity categories were compared "white" versus "Asian/other", whilst the category "other", in gender demographics, was not considered during the calculations.

A Mann–Whitney U test (*p* < 0.05) was performed, comparing the following characteristics: age, weight, height, smoking duration, time since quitting smoking and number of cigarettes smoked per day.

#### PAC-19QoL QoLIs validation

Using a Mann–Whitney test, statistically significant differences between the mean Likert score for each quality of life indicator (variable) were compared between the responses from study participants (both patients and control groups). A *p* value < 0.05 indicated a statistically significant finding in the presented analyses. All statistical analysis were conducted in R, version 3.6.3.

### PAC-19QoL validation

The PAC-19QoL instrument was validated using a control group with 16 healthy individuals recruited from the networks of the researchers. Participant demographics were recorded for the study patients and control populations, and they were required to complete the same questionnaire as the participants with a confirmed COVID-19 test. In addition, data generated from the control group were analysed using a Mann–Whitney test. This approach of using healthy volunteers and the Mann–Whitney test to validate a disease-specific quality of life instrument is a widely used scientific approach [19–22].

### Public and patient involvement

Through our ongoing work, we have established extensive networks with stakeholder groups and service users with rare diseases (such as XLH). In developing this project, we informally discussed the idea of developing a quality of life measure for people with a lived experience of COVID-19 with people within our extensive network. In total, the project idea was discussed with seven people, and each of these people expressed a need for a patient-centred Quality of Life measure that is easy to use and applicable to every aspect of their life, beyond the disease.

## Results

### Description of study participants

For the development of the PAC-19QoL, 15 long COVID patients were recruited; four (27%) and 11 (73%) were male and female, respectively. The average age of the study patients and control groups were 40 and 35, respectively (*p* = 0.308). In order to validate the PAC-19QoL, 16 unaffected participants were recruited. Nine (56%) of these were male, six (38%) were female, and one (6%) preferred not to say.

On assessing all baseline characteristics for heterogeneity, statistically significant differences were found between the two groups in the following demographics: long COVID symptoms (*p* < 0.001), COVID-19 in the past (*p* < 0.001), allergies (*p* < 0.05), asthma (*p* = 0.007), sleeping difficulty (*p* = 0.007), prescribed medications (*p* < 0.001) and dietary habits (*p* < 0.001). The study and control groups were comparable for all other demographic characteristics (Table 1). For all the analyses run in this study, a *p* value of 0.001 (Chi Square test) has been established, based on study participants experience of COVID-19 symptoms.

**Table 1:** Demographic characteristics of both control and study groups in the study

Characteristics	Control (n = 16)	Patients (n = 15)	*p* value
Male (n (%))	9(56)	4(27)	0.113^1^
Female (n (%))	6(38)	11(73)	
Other (n (%))	1(6)	0(0)	
Age (mean/years)	35	40	0.308^2^
Weight (mean/kg)	68	74	0.170^2^
Height (mean/cm)	173	166	0.054^2^
*Ethnicity (n (%))*			
White	9(56)	11(73)	0.699^1^
Asian	3(19)	1(7)	
Other	4(25)	3(20)	
*Country (n (%))*			
UK	3(19)	9(60)	
France	1(6)	0(0)	
Portugal	6(38)	0(0)	
India	2(13)	0(0)	
Germany	1(6)	0(0)	
Morocco	3(19)	3(20)	
Canada	0(0)	1(7)	
Ireland	0(0)	1(7)	
Belgium	0(0)	1(7)	
*Medical history (n (%))*			
long COVID symptoms	0(0)	15(100)	< 0.001*^1^
COVID-19 in the past	0(0)	15(100)	< 0.001*^1^
Organ transplant	0(0)	0(0)	NA
Major surgeries	1(6)	2(13)	0.953^1^
Cancer diagnosis	0(0)	0(0)	NA
Cystic fibrosis	0(0)	1(7)	0.974^1^
Asthma	0(0)	7(47)	0.007*^1^
COPD	0(0)	0(0)	NA
Diabetes: type 1	0(0)	0(0)	NA
Diabetes: type 2	6(38)	0(0)	NA
High cholesterol	1(6)	2(13)	0.436^1^
High blood pressure	0(0)	2(13)	0.953^1^
Stroke	0(0)	0(0)	NA
*Family history (n (%))*			
Cystic fibrosis (family history)	0(0)	1(7)	0.974^1^
Asthma (family history)	2(13)	3(20)	0.937^1^
COPD (family history)	0(0)	2(13)	0.436^1^
Diabetes: type 1 (family history)	0(0)	1(7)	0.974^1^
Diabetes: type 2 (family history)	6(38)	2(13)	0.260^1^
High Cholesterol (family history)	2(13)	5(33)	0.587^1^
High blood pressure (family history)	2(13)	4(27)	0.339^1^
Stroke (family history)	2(13)	3(20)	0.937^1^
*Drug history (n (%))*			
Prescribed medications	0(0)	10(67)	< 0.001*^1^
Non-prescribed or homoeopathic medications	1(6)	4(27)	0.291^1^
Immunosuppressant drugs	0(0)	1(7)	0.974^1^
Allergies	2(13)	8(53)	0.041*^1^
Pregnancy (n (%))	0(0)	0(0)	NA
*Social history (n (%))*			
Assistance with self-care needs	0(0)	1(7)	0.974^1^
Mobility issues	0(0)	1(7)	0.974^1^
Does own shopping	16(100)	12(80)	0.203^1^
Current smoker	2(13)	0(0)	0.494^1^
Former smoker	4(25)	3(20)	0.499^1^
Physical activity	16(100)	12(80)	0.203^1^
*Sleep history (n (%))*			
Sleeping difficulty	3(19)	12(80)	0.007*^1^
Falling asleep difficulty	3(19)	7(47)	0.446^1^
Staying asleep difficulty	2(13)	9(60)	0.169^1^
Sleep apnoea	2(13)	3(20)	0.358^1^
*Dietary habits (n (%))*			
Healthy	2(13)	12(80)	< 0.001*^1^
Unhealthy	14(88)	3(20)	
*Weight category (n (%))*			
Underweight	0(0)	1(7)	0.203^1^
Average	16(100)	12(80)	
Overweight	0(0)	2(13)	
*Employment (n (%))*			
Part time	0(0)	4(27)	
Full time	13(81)	6(40)	
Self-employed	3(19)	0(0)	
Unable to work	0(0)	1(7)	
Homemaker	0(0)	1(7)	
Student	0(0)	1(7)	

^1^Chi-Square *p* value

^2^Mann–Whitney U test *p* value

*p value < 0.05 is considered statistically significant

### Generation of quality of life indicators (variables) for inclusion in the PAC-19QoL instrument

The study included 15 participants with long COVID-19, and saturation of unique quality of life indicators was achieved by participant 9 (Appendix 2). Forty-nine unique indicators were generated during the Awareness Round (1) of the Jandhyala Method and grouped into the following four domains and 19 subdomains. These are:

1. Psychological (Mood, Isolation, Motivation, Anxiety, Cognition, Expression, Mental Exertion),
2. Physical (Exertion, Pain, Travel, Somnolence, Smell/taste, Breathlessness, Fine motor, Libido),
3. Social (Isolation, Relationships, Hobbies), and
4. Work (Ability to work)

**Fig. 1:**
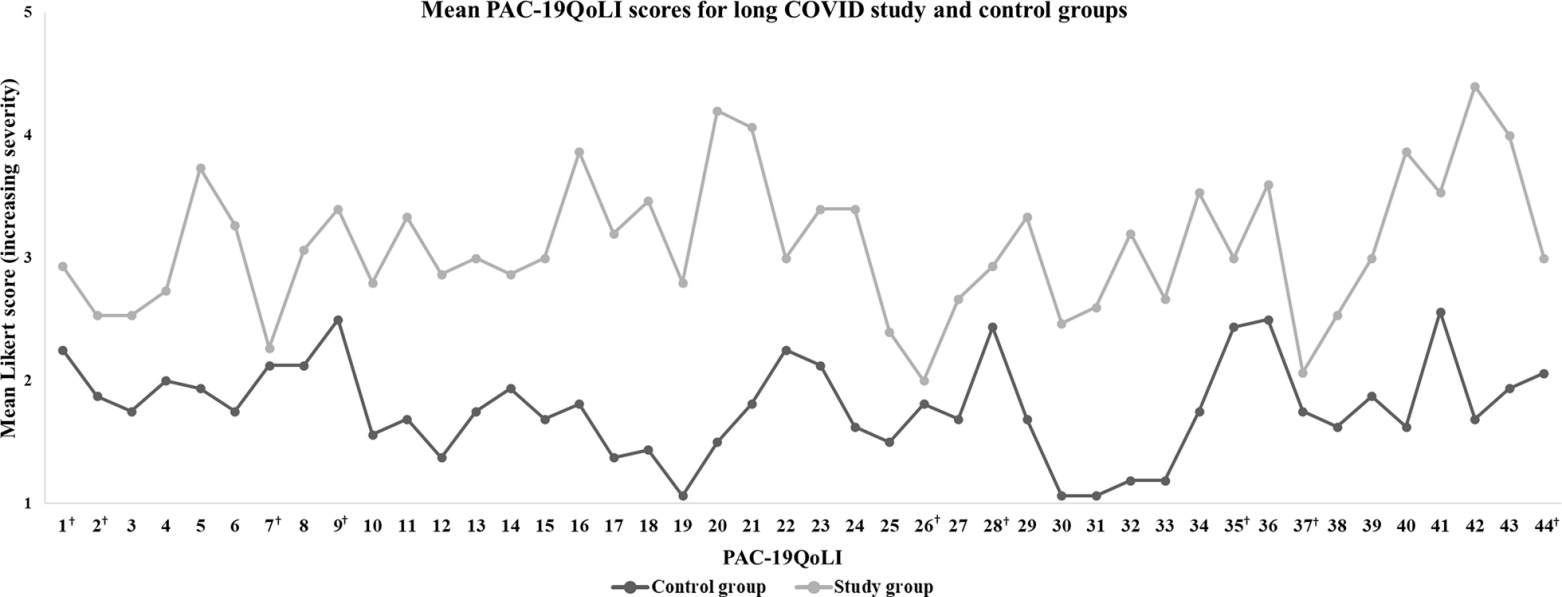
PAC-19QoLI Likert scores for control and study groups. ^†^QoLIs with no statistically significant differences between groups

Anxiety (Psychological domain) and Exertion (Physical domain) were the two most-populated subdomains, containing 16.3% (8/49) and 14.3% (7/49) of QoLIs, respectively. Of the 49 variables (QoLIs), 8 (16.3%) displayed an AI > 0.50. When the full list was presented to the participants in the Consensus Round (2), 48/49 (98%) achieved a relative degree of prompting. Since five QoLIs failed to reach the cut-off point of CI > 0.50, the remaining 44 QoLIs were included in PAC-19QoL. Validation of the PAC-19QoL instrument with regards to its specificity towards patients with long COVID showed that nine out of the 44 QoLIs failed to demonstrate a statistically significant difference between the patient and control groups (Table 2 and Fig. 1). The full list of 44 QoLIs was then converted to variables to populate the finalised PAC-19QoL instrument.

**Table 2:** The 44 QoLIs meeting threshold CI > 0.05 (CS:1&2) included in the PAC-19QoL and the Mann–Whitney test of difference in Likert means for each item between the study and control populations

Domain	Subdomain	QoLI	QoLI Title and Operational Definition	Score		Mean Likert Score		*p *value^1^
				A	C	Control	Patients	
Psychological	Mood	1†	Feelings of low mood (including tearfulness)	3	2	2.25	2.93	0.075
		2†	Feelings of anger	3	2	1.88	2.53	0.065
	Isolation	3	Feeling of being isolated or lonely due to the effects of long COVID	2	2	1.75	2.53	< 0.05*
	Motivation	4	Motivation to perform regular activities	3	2	2.00	2.73	< 0.05*
	Anxiety	5	Anxious about the future health of myself	3	2	1.94	3.73	< 0.001*
		6	Anxious specifically about falling ill with COVID-19 again	3	2	1.75	3.27	< 0.05*
		7†	Anxious about the future health of my children	3	2	2.13	2.27	0.693
		8	Anxious about the future and the quality of my relationships with my family	3	2	2.13	3.07	< 0.05*
		9†	Anxious about the future and my financial situation	3	2	2.50	3.40	0.085
		10	Anxious about being dependent on or a burden to relatives or others (such as charities)	3	2	1.56	2.80	< 0.05*
		11	Anxious for no apparent reason	3	2	1.69	3.33	< 0.05*
		12	Anxious about experiencing flashbacks of traumatic events	3	2	1.38	2.87	< 0.05*
	Cognition	13	Ability to process information and organise thoughts	2	2	1.75	3.00	< 0.001*
		14	Ability to focus/concentrate and perform house chores	3	2	1.94	2.87	< 0.05*
	Expression	15	Ability to express oneself verbally	3	2	1.69	3.00	< 0.001*
	Mental exertion	16	Tiredness due to mental exertion	3	2	1.81	3.87	< 0.001*
		17	Mental exertion triggers an immediate headache	3	2	1.38	3.20	< 0.001*
		18	Mental exertion triggers post-exertion malaise	3	2	1.44	3.47	< 0.001*
Physical	Exertion	19	Exacerbation of chest pain due to physical exertion	3	2	1.06	2.80	< 0.05*
		20	Exacerbation of breathlessness due to physical exertion	3	2	1.50	4.20	< 0.001*
		21	Tiredness due to physical exertion	3	2	1.81	4.07	< 0.001*
		22	Impact in the ability to perform daily chores due to exhaustion	3	2	2.25	3.00	< 0.05*
		23	Impact on the ability to participate in social events due to exhaustion	3	2	2.13	3.40	< 0.001*
		24	Ability to look after spouse or children	3	2	1.63	3.40	< 0.001*
		25	Ability to wash and dress	3	2	1.50	2.40	< 0.05*
	Pain	26†	Pain in joints	3	2	1.81	2.00	0.71
	Travel	27	Confidence in ability to drive	3	2	1.69	2.67	< 0.05*
		28†	Ability to travel using public or transport	3	2	2.44	2.93	0.066
	Somnolence	29	Feeling sleepy throughout the day	2	2	1.69	3.33	< 0.001*
	Smell/taste	30	Inability to enjoy food due to not being able to smell or taste it	3	2	1.06	2.47	< 0.05*
		31	Inability to enjoy alcohol due to not being able to smell or taste it	3	2	1.06	2.60	< 0.05*
	Breathlessness	32	Effect on the ability to converse due to breathlessness	2	2	1.19	3.20	< 0.001*
	Fine motor	33	Inability to carry out delicate tasks with fingers	2	2	1.19	2.67	< 0.001*
	Libido	34	Interest in sexual intercourse	2	2	1.75	3.53	< 0.05*
Social	Isolation	35†	Physical isolation from family members due to long COVID	1	2	2.44	3.00	0.278
		36	Physical isolation from friends due to long COVID	3	2	2.50	3.60	< 0.05*
	Relationships	37†	Relationship with spouse or partner	3	2	1.75	2.07	0.448
		38	Relationship with dependents	3	2	1.63	2.53	< 0.05*
		39	Relations with colleagues and friends	3	2	1.88	3.00	< 0.05*
		40	Exhaustion due to emotional exertion	3	2	1.63	3.87	< 0.001*
	Hobbies	41	Engaging normal hobbies or recreational activities	3	2	2.56	3.53	< 0.05*
Work	Ability to work	42	Current ability to work versus pre-COVID-19 state	3	2	1.69	4.40	< 0.001*
		43	Impact in career progression due to being ill or on sick leave	3	2	1.94	4.00	< 0.001*
		44†	Loss of income due to inability to work	3	2	2.06	3.00	0.14

^1^Mann–Whitney U test *p* value

^†^QoLIs with no statistically significant differences between groups

*Statistically significant at 95% confidence interval

## Discussion

The PAC-19QoL instrument for the assessment of the quality of life in patients with long COVID, was developed using the Jandhyala method to observe consensus on quality of life indicators solicited in response to the questioning of recruited patients on how long COVID has affected their quality of life. It also provided an insight into the distribution of quality of life indicators in their overall initial awareness among the study group and the final consensus. In this regard, 44/49 (89.98%) indicators were observed to have been prompted from below the awareness threshold to above the consensus threshold and therefore deemed appropriate for inclusion in the PAC-19QoL.

This high rate of prompting may reflect the limited ability of the participants to engage with the initial open-ended question due to the increased mental exertion involved in reviewing the areas of their life affected and then providing indicators. In contrast, the consensus round can be argued as a less intensive exercise with the subject being required to select a level of agreement on a pre-populated list. Understanding the intensely limiting impact long COVID has on cognition and mentation can help inform approaches to engaging with this patient population with information. This impact has been highlighted by other studies conducted among patients affected by COVID-19 [23, 24].

Perhaps unsurprisingly, the most populated subdomains were: Psychological > Anxiety and Physical > Exertion. The latter, along with the Psychological > Mental Exertion subdomain, is consistent with the reporting of post-exertional malaise in response to both physical and mental exertion by other researchers [23]. The former brings into focus the specific concerns long COVID generates in its sufferers on their future. Findings of post-traumatic stress disorder (PTSD) and idiopathic anxiety informs targets for supportive psychiatric, psychological interventions among a portfolio of multidisciplinary management strategies. The 44 quality of life indicators (variables) reaching the consensus threshold for inclusion in the final list of the PAC-19QoL instrument used the basic 5-point Likert scales to assess the severity of each indicator at the time of administering.

Long COVID-19, is a multisystem disease characterised by a range of symptoms, (disease indicators experienced by the patient) and clinical signs (disease indicators observed or elicited by the clinician) aligned to each of these body systems. In this study, some of the included quality of life indicators were observed to align with a body system through a potential aetiological link. For example, QoLI 32 (Table 2, ‘Effect on the ability to converse due to breathlessness), while identified in this study as a physical domain indicator, may have clinical origins in both the respiratory and cardiovascular systems [25]. Similarly, QoLIs 16,17 and 18 on mental exertion (Table 2, Psychological domain) have similar clinical associations with the neurological system. This being said, some included QoL indicators do not readily align to these body systems, most notably those in the social and work domains. This emphasises not only the fundamental differences in conceptualisation of QoL and clinical constructs but also the need to consider them separately, as not all of the clinical symptoms and signs associated with Long COVID-19 will impact sufferers’ quality of life and not all the QoLI’s will clinically manifest themselves [26]. Thus, QoL is a highly relevant but discrete construct that should be measured in Long COVID-19 patients to fully appreciate the impact of the disease with Neutrality.

On completion of its validation, the PAC-19QoL was implemented in the associated PAC-19QoLReg. This patient registry is currently recruiting and will observe a cohort of participants with long COVID, including those with a clinical diagnosis without a confirmed test, over a period of 12 months, with the PAC-19QoL being administering on a monthly basis. It offers the opportunity to track the quality of life of the participants with long COVID beyond simple symptom monitoring, although any relapsing or remitting characteristics will add valuable knowledge to this emerging and debilitation disease.

## Future work

The PAC-19QoL instrument as a disease-specific tool is a reliable tool that can be used to effectively measure the impact of long COVID on the quality of life of affected patients. There is need for future work to use the tool in different countries, across cultures, and different languages. This is likely to provide further insight into the validity and reliability of the PAC-19QoL instrument.

## Limitations of the study

An initial challenge to the recruitment of subjects to this study was the requirement for a positive test to ensure the indicators generated could be reasonably attributed to long COVID. This requirement generated a degree of frustration in potential participants, as a key concern around their initial management was the strict instructions of not presenting at their primary or secondary care facilities, thereby negating access to any form of testing.

A second potential limitation can be addressed by the fact that a number of QoLIs failed to differentiate between the affected and non-affected individuals. These indicators perhaps infer broader concerns around long COVID not limited to sufferers. Furthermore, these may relate to the general impact of the restrictions imposed to control the spread of the virus, e.g., 'physical isolation from family members' and 'ability to use public transport'. Quality of life indicators such as 'low mood' and 'anger', 'anxiousness about future health of children' and 'future financial situation' are understandable concerns for anyone living through a global pandemic of a novel virus.

A debate over whether the inclusion or exclusion of the quality of life indicators impacts the validity of the PAC-19QoL may ensue. While exclusion will remove any commonality of QoLIs between groups, thus reducing a false-positive rate, removing relevant QoLIs is likely to increase the false-negative rate. Given the overall proportion of these 9/44 (20%) and the damaging effect of not detecting and following up an individual with long COVID, a reasonable justification can be made for retaining them.

Another potential limitation is the number of participants, Although the numbers of participants included in the study were sufficient to test univariate associations, studies with larger number of participants are required to test multivariable associations towards confirming these results.

Finally, there is a potential for bias in the QoLIs included in the PAC-19QoL instrument. This due to patient characteristics, particularly the difference in the educational status of patients, and how this might affect the quality of their responses and contribution to the unique quality of life indicators generated during the Awareness Round (1) of the Jandhyala Method.

## Conclusions and implications

It is hoped that the successful development and validation of the PAC-19QoL, a long COVID disease-specific quality of life instrument and its implementation in a dedicated patient registry (PAC-19QoLReg) will complement ongoing research initiatives in monitoring long COVID QoL progression. It is also hoped that the development of the instrument will help to detect responses to therapeutic interventions with greater accuracy, ultimately informing patient care and improving outcomes.

## Data Availability

Data from this study will be made available upon reasonable request to the author.

## Appendix 1: Awareness round one (AR1) survey instructions

### Instructions to question one

As part of research studies, participants are often asked questions relating to their quality of life, and there are many questionnaires available for this. Currently, there are no post-COVID-19-specific questionnaires. As this is a new and emerging disease, we have yet to gather much information about how people’s lives are impacted in the months following a COVID-19 diagnosis. We are interested in finding out what you, as someone who has had COVID-19, think is important when measuring the quality of your life after this disease. This information will be used to help us design a questionnaire to be given to post-COVID-19 patients to measure the quality of their lives.

Often people do not think much about the areas of their lives which contribute to the quality of their lives; they might only notice that something was important when it changes or is going badly. We would like you to think carefully about how different aspects of your life impact on you day to day, and particularly tell us about any areas which have been impacted by COVID-19. We would like you to give us as much specific detail as possible, including examples, if you have them, of areas which have been affected for you. You can complete up to 50 boxes per category (please do not be overwhelmed by this number, we just wanted to make sure we gave you space to tell us about as many things as you want to) and we ask that you please try to complete at least 3.

If you have any questions about any of this or would like any support to complete this questionnaire, then please contact us at info@medialis.co.uk leaving details of your preferred way to be contacted and suitable times to do so. A member of the research team will be in touch to assist you as soon as possible.

Thank you for taking the time to help us with this research

### Instructions to question two

We have labelled the sections below with categories we think might be relevant to your quality of life, and have given you some text boxes to write in the things you think are important under each of these headings. You can complete up to 5 boxes per category, and we ask that you please try to complete at least 3. There is also a section for you to tell us about any other areas you think are important which we may have missed. We would like you to think carefully about how different aspects of your life impact your day to day activities, and particularly to tell us about any areas which have been impacted by COVID-19. We would like you to give us as much specific detail as possible, including examples if you have them of areas which have been affected for you. It might be helpful to think about the various things which help to make these areas of your life good or not so good and to think about what would happen if things changed within them to work out what is important to you.

The areas of your life might broadly fall into categories around:

- Mental wellbeing – anything which has an impact on your general mental wellbeing. Physical wellbeing- anything which has an impact on your physical wellbeing and health.
- Social wellbeing – anything which has an impact on your ability to have a social life with friends and family and your engagement with wider society.
- Material wellbeing – anything which has an impact on your ability to live comfortably and have the material things in life you need. Recreational wellbeing – anything which has an impact on your ability to engage in activities during your free time.
- Professional wellbeing – anything which impacts on your education, job, or career
- Overall wellbeing (balance) – anything which impacts on your ability to balance the various areas of your life Other.

## Appendix 2

These data present the emergence of unique PAC-19QoL Indicators, by each additional participant. They are provided in the context of the anticipated saturation of indicators with the pre-specified sample size.

The rate of accrual of new unique indicator is displayed as the absolute rate by bar graph and as the relative rate by the cumulative rate curve for each additional participant. It is proposed that the rate of addition of unique indicators is negligible beyond the 9th participant and saturation had been reached with this population of participants in this study.

This empirical observation confirms the number of participants required to accumulate a definitive set of qualitative indicators towards a specific research question when applying the Jandhyala Method.

## Abbreviations

AI: Awareness index
ARDS: Acute respiratory distress syndrome
CI: Consensus index
COPD: Chronic obstructive pulmonary disease
COVID-19: SARS-CoV-2 2019
PAC-19QoL: Post-acute (long) COVID-19 quality of life
PAC-19QoLReg: Post-acute (long) COVID-19 quality of life patient registry
PTSD: Post-traumatic stress disorder
QoL: Quality of life
QoLI: Quality of life indicators

## Declarations

### Transparency declaration

Medialis continues to run the PAC-19QoLReg patient registry in association with the Centre for Pharmaceutical Medicine Research, Institute of Pharmaceutical Science, Faculty of Life Science & Medicine, King's College London, as part of its Corporate Social Responsibility Project.
